# Patient participation in postoperative care activities in patients undergoing total knee replacement surgery: Multimedia Intervention for Managing patient Experience (MIME). Study protocol for a cluster randomised crossover trial

**DOI:** 10.1186/s12891-016-1133-5

**Published:** 2016-07-18

**Authors:** Jo McDonall, Richard de Steiger, John Reynolds, Bernice Redley, Patricia Livingston, Mari Botti

**Affiliations:** School of Nursing and Midwifery, Deakin University, Geelong, Australia; Surgery Epworth HealthCare, The University of Melbourne, Parkville, Australia; Faculty of Medicine, Nursing and Health Sciences, Monash University, Clayton, Australia; Faculty of Health, Deakin University, Geelong, Australia; Epworth/Deakin Centre for Nursing Research, Epworth HealthCare, Melbourne, Australia

**Keywords:** Patient participation, Patient involvement, Patient experience, Patient satisfaction, Acute care, Cluster randomised crossover trial, Multimedia intervention, Health service evaluation

## Abstract

**Background:**

Patient participation is an important indicator of quality care. Currently, there is little evidence to support the belief that participation in care is possible for patients during the acute postoperative period. Previous work indicates that there is very little opportunity for patients to participate in care in the acute context. Patients require both capability, in terms of having the required knowledge and understanding of how they can be involved in their care, and the opportunity, facilitated by clinicians, to engage in their acute postoperative care. This cluster randomised crossover trial aims to test whether a multimedia intervention improves patient participation in the acute postoperative context, as determined by pain intensity and recovery outcomes.

**Methods/design:**

A total of 240 patients admitted for primary total knee replacement surgery will be invited to participate in a cluster randomised, crossover trial and concurrent process evaluation in at least two wards at a major non-profit private hospital in Melbourne, Australia. Patients admitted to the intervention ward will receive the multimedia intervention daily from Day 1 to Day 5 (or day of discharge, if prior). The intervention will be delivered by nurses via an iPad™, comprising information on the goals of care for each day following surgery. Patients admitted to the control ward will receive usual care as determined by care pathways currently in use across the organization. The primary endpoint is the “worst pain experienced in the past 24 h” on Day 3 following TKR surgery. Pain intensity will be measured using the numerical rating scale. Secondary outcomes are interference of pain on activities of daily living, length of stay in hospital, function and pain following TKR surgery, overall satisfaction with hospitalisation, postoperative complications and hospital readmission.

**Discussion:**

The results of this study will contribute to our understanding of the effectiveness of interventions that provide knowledge and opportunity for patient participation during postoperative in-hospital care in actually increasing participation, and the impact of participation on patient outcomes. The results of this study will also provide data about the barriers and enablers to participation in the acute care context.

**Trial registration:**

Australian New Zealand Clinical Trials Registry ACTRN12614000340639 Trial Registration date 31/03/2014.

## Background

Active participation of patients in their care is important for ensuring safe and high quality healthcare [1]. Factors known to impact on patients’ capability and opportunity to participate in healthcare include both patient and nurse-related factors. Patient-related factors that impact on their ability to participate in healthcare include acceptance of the new patient role [2], medical knowledge [3–5], level of confidence [6, 7], knowledge related to condition [8, 9], acuity of illness, and comorbidity [2, 10, 11]. For nurses, acceptance and promotion of patient participation is influenced by issues such as the desire to maintain control [7, 12, 13], the acuity of patients’ illness [2, 14] and available time [14].

In Australia, total knee replacement (TKR) surgery is an increasingly common treatment for patients with osteoarthritis [15]. TKR surgery is performed to reduce knee pain, improve function and to improve quality of life [16]. In 2013, 48,502 knee replacements were undertaken nationally [15]. This represents an increase of 2.7 % compared to numbers reported in 2011. The majority of TKR surgeries in 2012 (76.9 %) were in private hospitals [15]. Of these, 21.5 % (*n* = 10,435) were performed in Victoria [15]. TKR surgery is a relatively common and successful procedure, but also considered one of the most painful [17] postoperatively.

In order for patients to achieve maximum benefit from TKR surgery they must commence early mobilisation of the knee joint to maximise range of movement [18]. Adequate pain management is fundamental to achieve early mobilisation and meet postoperative goals of recovery. High levels of postoperative pain intensity are linked to reduced knee mobility and prolonged recovery times [19, 20]. Poorly controlled pain postoperatively has also been associated with the development of ongoing chronic pain [21].

The complexity of the acute postoperative environment and severity of symptoms after TKR surgery may limit patients’ participation in their care, leading to variability in the quality of pain management they receive [22], and subsequently, their ability to meet postoperative recovery goals. Pain is a subjective experience [23] and in order for nurses to understand patients’ level of pain and provide appropriate interventions, patients need to be involved and actively participate in the control of their pain management [24]. Variability in the quality of postoperative pain management in the context of joint replacement surgery can have significant consequences for patients, including poor overall recovery [25], reduced physical functioning, increased length of stay [26] and an unsatisfactory patient experience [27].

To date, the majority of studies examining patient participation in their care have focused on patient participation in medical treatment decisions and self-management associated with chronic life-long illness [9, 12, 28]. Patient participation in meeting treatment goals of care during acute episodic illness is poorly understood and requires further exploration. The current study addresses this gap by using an intervention designed to facilitate patient participation in achieving adequate pain management and therefore meet the goals of recovery in the immediate postoperative period after TKR surgery. A nurse-led, multimedia animation intervention will be implemented immediately after surgery in order to increase both the capability of (through information) and opportunity for (through interaction with nurses) patients to participate in their goals of care after TKR surgery.

### Aims of the study

The purpose of this research is to design, implement and evaluate a nurse-led multimedia education intervention to improve postoperative outcomes for patients post TKR and explore the relationships between patient participation, patient experience and patient satisfaction with care.

The overall aim of this study is to test whether a nurse-led, multimedia intervention, designed to provide knowledge and opportunity for patients to participate in their acute postoperative recovery after surgery, improves their recovery.

## Methods/Design

**Fig. 1:**
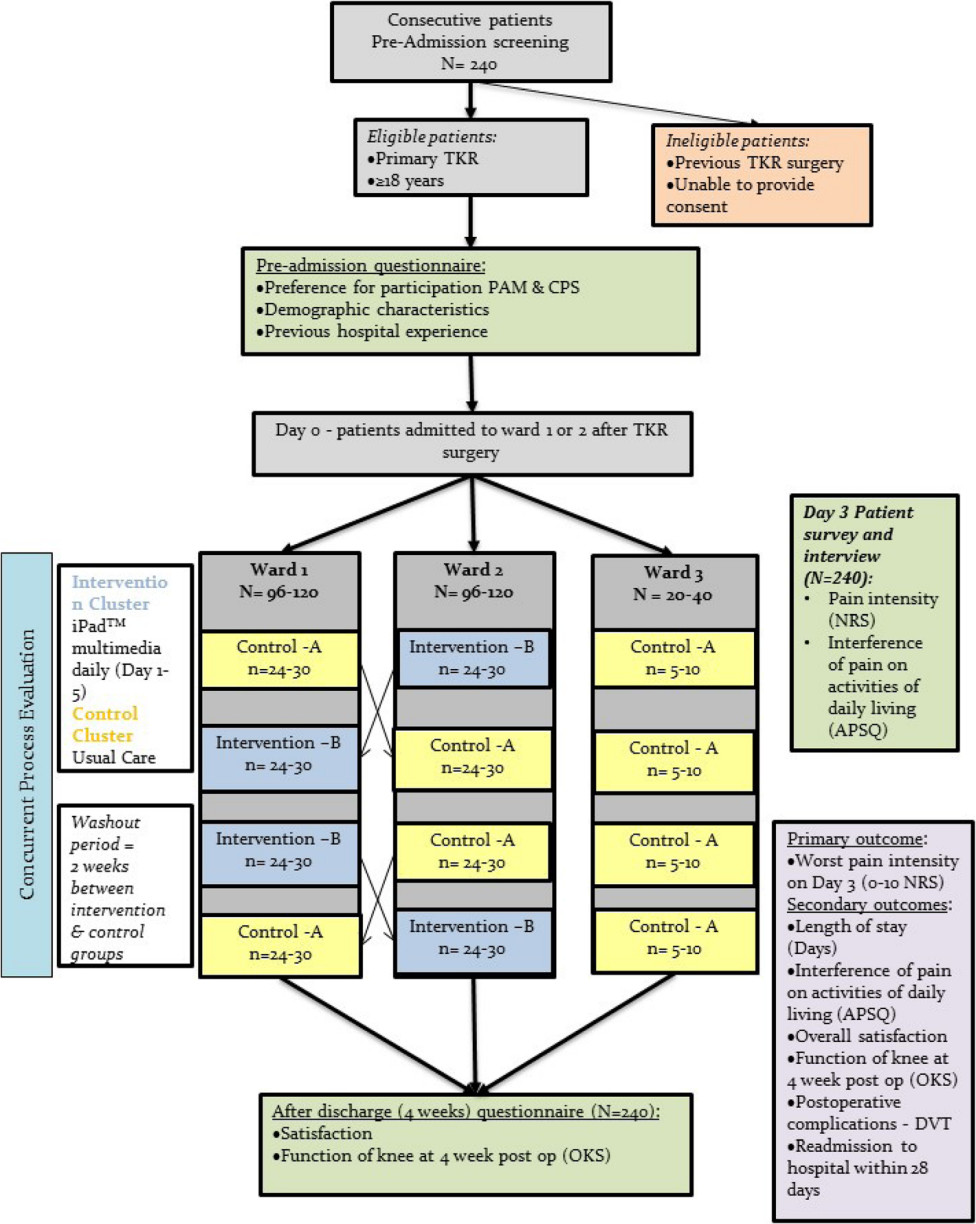
Study design

A cluster randomised, crossover trial and simultaneous process evaluation will be undertaken using mixed-methods for data collection and analysis. In this study, each cluster refers to a hospital ward (1, 2 or 3) and the cohort refers to the group of patients admitted to the ward during a specified period of time. Two clusters, or wards, will receive both the intervention and control (usual care) conditions in separate time periods, and, in sequences that are randomised to the two wards. The third ward will act as an “overflow” ward for patients who are consented on the study but are unable to be admitted, when the wards are full, to either of the two orthopaedic wards that received randomized sequences of conditions. This third ward will receive the control condition only as we are unable to guarantee that this ward will contain 20 to 30 consented patients in a period and so assigning the intervention to this ward would have been wasteful (in terms of sample size allocation). The trial can be described as a cluster randomised crossover design [29] with two clusters and an added control cluster. The two crossover wards will be randomised to sequences of conditions to ensure that at least one ward receives the intervention and the other ward receives the control in each period. See Fig. 1 for an illustration of the study design.

### Setting

The study will take place in one Victorian non-profit private health service.

### Participants

Patients undergoing primary Total Knee Replacement (TKR) surgery are a high volume patient group at the participating health service. Patients after TKR who participate in their postoperative management are highly likely to benefit from participation in their postoperative recovery and these patients also have a high incidence of high intensity postoperative pain [20].

Inclusion criteria*:* Adults, aged 18 years or more, with a planned elective admission for primary TKR surgery, who are able to provide written, informed consent and complete English language questionnaires.

### Recruitment of patient participants

Recruitment will take place prior to surgery to allow potential participants time to reflect and seek clarification of their proposed involvement as well as to allow for baseline data collection. Recruitment of patient participants will take place using the method described below:

#### Method – preadmission clinic recruitment

At the participating hospital, the usual practice is the preadmission clinic coordinator contacts all patients scheduled for TKR prior to their attendance at the clinic and/or admission for surgery. At this time the coordinator will explain to patients they may be approached at the clinic about possible participation in a research study.

Wherever possible a member of the research team will attend the clinics and liaise with the clinic coordinator to identify potential participants. Suitable patients will be approached by the researcher who will invite them to read a study information sheet. Those interested in participation will be provided with a Participant Information and Consent Form and the baseline questionnaire.

### Multimedia goals of care intervention

The multimedia program was developed specifically for TKR patients by Enlighten Health®, a medical multimedia production company specialising in validated content for patient and clinical education. The multimedia intervention is presented in a format that combines text, sound, graphics and animation to provide information to patients in relation to postoperative recovery and goals of care for each day, up to 5 days following a TKR.

The multimedia program intervention, is designed to be both nurse-led, where the nurse assists the patient to navigate through the program, and patient self-directed; it is delivered to patients as a stand-alone program package on an iPad™. The intervention is designed with tailored information about the expected postoperative recovery on each day from Day one (1) to Day five (5) (or day of discharge) after TKR surgery. Embedded throughout the program is the importance of adequate pain management in order to achieve daily goals of recovery. The aim of the intervention is to facilitate an interaction between patients and their nurse each postoperative day about the specific goals and plans of care for each day of recovery after TKR surgery. The three main goals of recovery for patients after TKR surgery are: 1) to manage pain, 2) promote mobility of the knee joint to improve function and 3) avoid complications such as thromboembolism [16].

#### Intervention application procedure

Daily, until discharge from the ward, following nursing handover and after patients’ morning meal (approximately 0900 h), the nurse allocated to care for a patient enrolled in the study reminds the patient to view the corresponding day’s goals of care package on the iPad™. The iPad™ is secured to the patient’s bedside to allow easy access to the program any time of the day or night and in addition, patients’ relatives can also view the presentation. It is anticipated that after viewing the goals of care animation, patients together with the nurse will discuss the patient’s goals for the day.

### Control – usual care

The control condition, received by a cohort of patients admitted to a ward over a particular time period, consists of usual care based on the clinical pathways currently in use across the organisation for patients after TKR surgery.

### Research setting

Data will be collected in three acute inpatient orthopaedic wards of a major, private, metropolitan hospital in Melbourne. The wards comprise a total of 79 acute orthopaedic beds and provide care for approximately three new patients undergoing a TKR per day on two wards with the third ward utilised as ‘overflow’. The units are staffed with teams of specialist nursing, medical and allied health staff to manage orthopaedic conditions.

## Methods and measures used for data collection from participants

### Pre admission

Consenting patients will be given a self-report questionnaire to complete and return pre-admission either via mail or handed back to the researcher present in the clinic. The concepts measured and the tools used in the pre-admission questionnaire are outlined in Table 1. This includes baseline characteristics such as age, sex, previous acute hospital experience and control preference; factors known to impact on patients’ level of participation and barriers to pain management.

**Table 1:** Concepts measured and tools used in Pre-admission Questionnaire

Concept measured pre admission	Tool used
1. Preference for participation	• Patient Activation Measure (PAM) (Cronbach *α* = 0.80 and 1.34) [28] • Control Preference Scale (CPS) [29]
2. Baseline characteristics	• Age, sex, previous hospital experience, cultural background, employment status
3. Patient barriers to management of pain	• Pain barriers questionnaire (PBQ) (Cronbach α = 0.73–0.83) [30]

### Inpatient data collection: cluster randomisation with crossover

Patients will be admitted to one of the orthopaedic wards (clusters) through normal hospital procedures.

The wards will have been randomly assigned to a sequence of control (A) and intervention (B). The intervention, “B”, will be allocated to at least four cohorts. The intervention appears once in each period and twice in each of the two wards. Ward 1 will use the sequence ABBA and on Ward 2 the sequence BAAB and Ward 3 will be assigned AAAA (Fig. 1). Each cohort within a cluster (data collection period) will be accrued and monitored over a period for 12–15 weeks. To mitigate any risk of contamination between control and experimental groups, a wash out period (usual care on all wards, no intervention or data collection) between cohorts will be of approximately 2 weeks in duration.

Participants admitted to the control wards (A) during data collection periods will receive usual care based on the clinical pathways for TKR recovery. In addition to usual care, patient participants admitted to the intervention (B) ward during data collection will receive the multimedia intervention (via iPad™) each day, commencing on Day 1 after surgery.

#### Day 3 – data collection

Data will be collected from each participating patient in a cohort of a ward (A or B conditions) on postoperative Day 3. On this day, patients will be interviewed by the researchers and invited to respond to questions about their pain intensity, interference of pain on activities of daily living and to describe processes of care and interactions with clinicians relating to their goals of recovery. In addition, patients on the intervention ward (B) will also be invited to respond to questions related to the intervention. Table 2 outlines the concepts, measures and tools used during this data collection period.

**Table 2:** Concepts measured and tools used for primary and secondary outcomes

Concept measured on day 3	Tool used
1. Pain intensity	• Numerical Rating Scale (NRS) [31]
2. Pain quality	• American pain society outcome questionnaire (APSOQ) (Cronbach α = 0.85) [32]
3. Pain treatment and management	• Medial record audit
4. Preference for participation	• Patient Activation Measure (PAM) • Control Preference Scale (CPS)

#### Medical record audit

Patients’ medical records will be audited on Day 3 to elicit analgesic administration over the 24 h period prior to the patient interview. The audit of pain treatment will involve review of medication charts to determine the type, dose and frequency of analgesic medications prescribed and the amount of analgesics administered as a ratio of administered verses available treatment in the 24 h period prior to interview.

### Follow up

A follow up questionnaire will be mailed to participants 4 weeks after they are discharged from the acute care ward. The concepts measured in this questionnaire include preference for participation (PAM & CPS), pain and functioning of knee after knee surgery (OKS), and patient satisfaction (NET promoter & global satisfaction) see Table 3. Patients will be asked to return the completed questionnaire using the pre-paid envelope provided. A reminder follow up telephone call approximately 2 weeks after the questionnaire mail-out will be made to remind patients to return the questionnaire.

**Table 3:** Concepts measured and tools used for post discharge Questionnaire

Concept measured on day 3	Tool used
1. Preference for participation	• Patient Activation Measure (PAM) [28] • Control Preference Scale (CPS) [29]
2. Pain and functioning of knee after knee surgery	• Oxford Knee Score (OKS)
3. Patient Satisfaction	• NET promotor and global satisfaction

Data related to complications and readmission to hospital (within 28 days of discharge) will be obtained through hospital information systems and chart audits.

### Tools used for data collection

#### Patient activation measure

The Patient Activation Measure (PAM) [29] is a 22-item questionnaire that assesses patients’ knowledge, skill, and confidence for self-management [30]. The short form version [6] used in this study, has 13-items measured in four stages: 1) believes active role is important, 2) confidence and knowledge to take action, 3) taking action, and 4) staying the course under stress.

#### Control preference scale

The control preference scale (CPS) is a five item ordinal scale to measure preference for treatment decision making role. Control preferences are defined as “the degree of control an individual wants to assume when decisions are being made about medical treatment” [31]. The CPS consists of five questions that each portrays a different role in treatment decision-making using a statement. Patients will be asked to rank their participation preferences in order from most preferred option to least preferred option. This tool will be used both preoperatively and postoperatively to identify patients’ preference for participation in postoperative activities during their hospitalisation at the time of discharge.

#### American pain society patient outcome questionnaire-revised

This is a validated instrument that has been widely used internationally [32]. The questionnaire is designed for use in adult hospital pain management quality improvement activities and American Pain Society’s Patient Outcome Questionnaire (APSOQ-R) measures six aspects of quality: (1) pain severity and relief; (2) impact of pain on activity, sleep, and negative emotions; (3) side effects of treatment; (4) helpfulness of information about pain treatment; (5) ability to participate in pain treatment decisions; and (6) use of non-pharmacological strategies.

#### Numerical rating scale

The numerical rating scale (NRS) [33] is a 0–10 point scale where patients are asked to rate their pain using a whole number ranging from 0 (no pain) to 10 (worst pain).

#### NET promoter scale

This scale is used to determine patients’ satisfaction with hospital care. This single item question elicits patients’ willingness to recommend the health care facility to family or friends on a scale of 0–10.

#### Oxford knee score

The Oxford Knee Score (OKS) is a 12-item patient self-report questionnaire specifically designed and developed to assess function and pain after total knee replacement (TKR) surgery. It is short, reproducible, valid and sensitive to clinically important changes [34]. Use of the tool is widely reported in research literature and has been adapted and validated in several languages [35–37].

### Process evaluation

The process evaluation is designed to examine the delivery of the intervention and understand the effects (or not) in the context of acute care delivery [38]. In order to know if the multimedia intervention was successful or not, in reducing patient participant pain intensity scores on Day 3, underlying processes need to be examined.

Process evaluation in this trial will include patient participant semi structured interviews on Day 3 post operatively of all participants, to examine patient barriers and facilitators to participation in care. Specific questions will be asked of participants relating to their goals of recovery in order to describe processes of care and interactions with clinicians. In addition to the interviews, field notes will also be kept by the researches to record information about ward culture and organisational environment that may impact on patients’ ability to participate.

Medication chart and medical records audits will be conducted on Day 3 to elicit information regarding prescribing and administration of analgesia. Furthermore medical records will be audited for documentation regarding pain though out the 24 h period on Day 3, this will give us details of the management and recording of patient participants’ pain.

### Outcome measures

The effectiveness of the multimedia intervention will be assessed by the following outcome measures:

1. The primary outcome is the difference in patients’ reported pain intensity (Numerical Rating Scale) on Day 3 between control and intervention group.
2. Secondary outcome measures include:
  Interference of pain on activities of daily living (APSOQ-R) on Day 3
  Length of hospital stay (days)
  Function and pain following TKR surgery (Oxford Knee Score) 4 weeks after discharge from acute care
  Patient overall satisfaction (NET promotor score) 4 weeks after discharge from acute care
  Postoperative complications – Deep Vein Thrombosis (within 28 days)
  Readmissions to hospital (within 28 days)
3. To conduct a detailed concurrent process evaluation to examine the delivery of the intervention and understand the effects of the intervention in activating patient participation in the context of acute care delivery [38]. Furthermore, the process evaluation will provide rich data relating to the significance of participation to patient participants, and barriers and facilitators to participation in this context.

### Rationale for pain as the primary endpoint

Pain intensity will be measured on Day 3 after TKR surgery using the NRS. Alleviation of pain is a fundamental obligation of healthcare providers, yet clinical studies continue to show that current practices to alleviate pain are unsatisfactory [39, 40]. Poor pain management is associated with serious physiological and psychological sequelae that compromise recovery and negatively affect morbidity and mortality [41]. Suboptimal treatment of acute postoperative pain is also strongly associated with the development of chronic pain [21, 42, 43]. Risk factors for developing chronic post-surgical pain include unrelieved acute pain, persistent severe pain, inappropriate use of analgesics following surgery, as well as patients’ pain-related beliefs [41, 43]. Patient participation in pain treatment decisions can positively influence their postoperative pain experience and lessen risk of progression to chronic pain [40] however studies show that patients have little opportunities to participation in pain management decisions [44].

### Primary endpoint

The primary endpoint for the assessment of patient experience is the “worst pain experienced in the past 24 h” on Day 3 following TKR surgery. Worst pain will be measured using the 0–10 NRS where 0 equates to “No pain” and 10 equates to “Worst possible pain”. Patients will be asked to choose a whole number from 0 to 10 that best describes their worst pain experienced in the previous 24 h.

### Sample size calculation

In this study, the null hypothesis (H_o_) associated with the primary objective is that no difference exists between patients in the intervention group and the control group in terms of worst pain score on Day 3 post TKR surgery. NRS pain intensity scores will be analysed via the fitting of a linear mixed model with random effects for clusters, cohorts within clusters, and, patients within cohorts, and, fixed effects for the conditions. The F-test, conducted at the 5 % significance level, will be used to compare the average pain scores for the two conditions (intervention versus control).

The number of clusters is essentially fixed (i.e. two or possibly three wards) but the number of periods, or cohorts per ward, (2, 3 or 4) and the number of patients in each cohort (24 or 30) was selected in order to achieve 80 % power when there are at least two wards and when the difference (delta) between the average pain scores for the two conditions is 1.65 and at least 70 % power when delta is 1.5. Preliminary (unpublished) data indicate that pain scores decline by 1 to 1.5 units from day 3 to day 4 post TKR surgery and so a delta of 1.5 to 1.65 at 3 days is a similar but enhanced improvement.

Pilot data results also indicated a between-patient (i.e. within-cohort) standard deviation equal to 2 (i.e. between-patient variance component, VP, equals 4) and a grand mean equal to 7. The between-ward variance component (VW) and between-cohorts variance component (VC) were assumed to be equal to 0.025 (this was based on unpublished data for two wards assessed in the same time period).

As the NRS pain intensity score is a bounded discrete outcome score, the power of the F-test was calculated by simulation. Numerical Rating Scale (NRS) observations were generated by adding normal random variables sampled from three distributions (corresponding to the three sources of variation and their specified variance components) to the conjectured fixed effect means and then rounding the result to the nearest whole number in the range of 0–10. In this way, the bounded and discrete nature of the NRS scores was accounted for in analyses of variance (ANOVA) designed for continuous scale variables. For each scenario (combinations of delta, variance components, cohort sizes and ranges of period effects) 10,000 simulations of each “study” were performed and the type II error rate (β) was calculated. The ANOVA directive and programming language in the GenStat statistical system (Release 14.2) were used to perform the simulations [45].

Results of the simulations when period effects are assumed to be equally spaced are shown in Table 4. When the range of the period effects decreases, the power increases. With two wards, the effect difference that would be detectable with 80 % power, when the period effects are in the range from -1 to +1, VW, VC and VP are 0.025, 0.025 and 4 respectively AND when the cohort size is 24, is delta = 1.80, and when the cohort size is 30, delta reduces to 1.65.

**Table 4:** Power and sample size calculations (based on two wards and four periods)

Range of period effects	Delta	VW	VC	VP	NP	Power
−1.5, 1.5	1.50	0.025	0.025	4.0	24	0.651
−1.0, 1.0	1.50	0.025	0.025	4.0	24	0.670
−0.75, 0.75	1.50	0.025	0.025	4.0	24	0.680
−1.5, 1.5	1.50	0.025	0.025	4.0	30	0.714
−1.0, 1.0	1.50	0.025	0.025	4.0	30	0.741
−0.75, 0.75	1.50	0.025	0.025	4.0	30	0.744
−1.0, 1.0	1.80	0.025	0.025	4.0	24	0.797
−1.0, 1.0	1.65	0.025	0.025	4.0	30	0.803

Footnote: Power and sample size calculations were calculated for the F test (α = 0.05) for a difference between the intervention and control groups in Day-3 worst pain. Delta is the absolute value of the difference in the mean pain scores. Components of variance in Day-3 pain scores are fixed as follows: between wards (VW = 0.025), between cohorts of patients within the same ward (VC = 0.025) and between patients within a cohort within a ward (VP = 4.0). NP is the number of patients in a cohort. The power (1-β) is the probability that the null hypothesis, of no difference in the mean Day-3 worst pain scores between the control and intervention groups, is rejected when the true, but unknown, difference is delta, the components of variance are as given, there are two wards and four cohorts per ward managed contemporaneously in four time periods, and the F-test is conducted at the 5 % significance level (α = 0.05). In these scenarios, three different ranges (in equally spaced steps) for the effects of the four periods on day-3 worst pain scores are investigated

Four periods and a target of 30 patients per cohort were selected (a total of 240 patients).

## Data analysis

### Quantitative analysis

Quantitative data obtained through patient questionnaires will be analysed using GenStat statistical system (Release 14.2) and verified using Statistical Package for the Social Science (SPSS V22). Statistical significance will be claimed at *p* <0.05. Descriptive statistics will be used to characterise the study population, and any baseline differences between patients in the control and intervention groups.

The primary endpoint of pain intensity will be compared between groups to determine differences between the intervention and control (usual care). A linear mixed model analysis will be used to calculate the F-test to compare the means of the groups. In a supportive analysis, the REML method will be used to estimate, and if necessary adjust for, period effects. Other outcome measures such as preference for participation, control preference and perceived participation in care will be used to make comparisons between groups and the analyses will use a linear mixed model approach and analogous methods developed for binary and categorical data.

### Qualitative analysis

Qualitative data obtained through questionnaires and patient interviews. Patient participant interviews will be tape recorded and transcribed in full. A critical step in this study relates to evaluating the intervention, i.e. whether the multimedia intervention changed the way patients interacted with clinicians in order to achieve their goals of recovery. This is a fundamental aspect of the process evaluation. The interview transcripts will be analysed using quantitative content analysis [46]. A structured, systematic coding scheme will be applied to the textual data in order to compare intervention and usual care content for each of the four goals of recovery addressing the five main concepts of:

1. Knowledge of the goal;
2. Engagement with achieving the goal;
3. Attitudes towards participation;
4. Perceived barriers to participation;
5. Perceived enablers to participation.

The unit of analysis will be text in the transcripts that refers to one of the four goals of recovery to be analysed. Several steps will be taken to conduct these analyses:

- The team will review 14 randomly selected transcripts (7 intervention and 7 non-intervention transcripts) to determine if the narrative could be relied on to provide sufficient information to conduct the analyses intended and identify coding rules for the five goals of recovery in the multimedia intervention.
- Coding rules will be developed in this process.
- These coding rules will be applied independently by all the investigators to assess the usability, ease and inclusiveness of the coding rules, and pilot test the inter-rater reliability of the coding rules on another randomly selected 10 transcripts.
- Once there is agreement about the coding process, a coding book will be developed and the remaining transcripts will be coded.
- The coding will be done by the researcher and an independent coder
- Once coding is completed, the research team will conduct inter-rater reliability measures including percentage agreement. Inter-rater agreement greater than 80 % is considered acceptable [47]
- Minor discrepancies between the coders will be resolved by examining the data together.
- Major discrepancies will be resolved by independent review by two other members of the research team.

## Trial status

Trial status at the time of manuscript submission is ongoing. The trial is currently in the last phase of data collection.

## Discussion

The purpose of this research program is to develop, implement and evaluate a nurse-led multimedia, education intervention to improve postoperative outcomes for patients having TKR surgery. Our previous work in relation to patient participation in acute care has shown very low levels of patient involvement in care. In order to understand the complexities of facilitating patient participation in recovery after surgery, interventions to improve the capability of patients to participate need to be developed and evaluated. Evidence of the effectiveness of interventions that influence patient participation, on patient outcomes in the acute postoperative context, is not available. The outcomes of this research will inform the vital aspects of patient involvement in care processes and importantly provide evidence for the effectiveness of participation in improving patient outcomes after surgery.

## Abbreviations

APSOQ-R, American pain society’s patient outcome questionnaire; CPS, control preference scale; NRS, numerical rating scale; OKS, Oxford knee score; PAM, patient activation measure; TKR, total knee replacement
